# Plasma ghrelin level and plasma ghrelin/obestatin ratio are related to intestinal metaplasia in elderly patients with functional dyspepsia

**DOI:** 10.1371/journal.pone.0175231

**Published:** 2017-04-18

**Authors:** Su Hwan Kim, Ji Won Kim, Junsu Byun, Ji Bong Jeong, Byeong Gwan Kim, Kook Lae Lee

**Affiliations:** Department of Internal Medicine, Seoul Metropolitan Government Seoul National University Boramae Medical Center, Seoul, Republic of Korea; Monash University, AUSTRALIA

## Abstract

**Background:**

Whether plasma ghrelin/obestatin levels are associated with *Helicobacter pylori* (*H*. *pylori*) infection, subtypes of functional dyspepsia (FD), and gastric mucosal histology has not yet been established in elderly patients.

**Objective:**

The aim of this study was to determine whether plasma ghrelin and obestatin levels are related to gastric mucosal histology, *H*. *pylori* infection, and FD subtypes in elderly patients with FD.

**Methods:**

Ninety-two patients diagnosed with FD and older than 60 years (median age 69.4; range 60–88) were included. Clinical symptoms investigated included postprandial fullness, epigastric pain, epigastric soreness, nausea, and vomiting. According to the Rome III criteria, patients diagnosed with FD were divided into two subtypes: epigastric pain syndrome (EPS) and postprandial distress syndrome (PDS). Plasma ghrelin and obestatin levels were measured using enzyme immunoassay, and histological examination of gastric mucosa was performed. *H*. *pylori* infection was determined by histopathological examination of gastric mucosal biopsy and/or Campylobacter-like organism test.

**Results:**

In our study, plasma ghrelin levels and plasma ghrelin/obestatin (G/O) ratio were significantly lower in subjects with intestinal metaplasia compared with those without intestinal metaplasia (ghrelin, p = 0.010; G/O ratio, p = 0.012). On the other hand, there were no significant differences in plasma ghrelin and obestatin levels between *H*. *pylori*–positive and *H*. *pylori*–negative groups. (ghrelin, p = 0.130; obestatin, p = 0.888). Similarly, no significant differences were detected between the EPS and PDS groups (ghrelin, p = 0.238; obestatin, p = 0.710).

**Conclusions:**

Patients with intestinal metaplasia, a known precursor of gastric cancer, had significantly less plasma ghrelin levels and G/O ratio than those without intestinal metaplasia.

## Introduction

Ghrelin is a 28-amino acid peptide which was discovered from the stomach and is known to be related to appetite regulation, satiety, and gut motility [1, 2]. The ghrelin gene consists of 4 exons and 3 introns. The major ghrelin gene products include acyl ghrelin, des-acyl ghrelin, des-Gln14-ghrelin, and obestatin [3]. Acyl ghrelin features a post-translational modification of O-n-octanoylation at serine 3 and has roles in food intake and energy homeostasis. Des-acyl ghrelin lacks O-n-octanoylation at serine 3, was reported to induce a negative energy balance by decreasing food intake and delaying gastric emptying [4]. Obestatin is a 23-amino acid peptide which is known to inhibit gastroduodenal motility in the fed state [5]. Obestatin is produced from the same precursor that produces ghrelin, and is secreted mainly from the stomach [6]. Studies on the relationship between functional dyspepsia (FD) and ghrelin reported that circulating ghrelin levels are decreased particularly in patients with postprandial distress syndrome (PDS) or dysmotility-like FD [7–9]. These patients were known to have low preprandial ghrelin levels and no significant decrease in ghrelin postprandially [8, 9]. However, the association between ghrelin levels and subtypes of FD has not yet been established.

In Gao et al’s study, plasma ghrelin levels and ghrelin/obestatin (G/O) ratio were lower in patients infected with *Helicobacter pylori (H*. *pylori*) [10]. *H*. *pylori* infection causes chronic gastritis, leading to gastric atrophy and intestinal metaplasia, which are precursors of gastric cancer [11]. However, in a study of patients without atrophic gastritis, *H*. *pylori* did not influence circulating ghrelin levels [12]. In addition, another study by Gao et al showed plasma ghrelin levels and G/O ratios were decreased in atrophic gastritis than in healthy subjects [13]. Thus, gastric atrophy rather than *H*. *pylori* infection has been suggested to be more related to circulating ghrelin levels. However, there has been no report on the association of circulating ghrelin, obestatin levels and other histological finding like intestinal metaplasia, a known histological precursor of gastric cancer [14]. Infection with *H*. *pylori* can lead to gastric atrophy and intestinal metaplasia [11]. The association between plasma ghrelin levels and *H*. *pylori* infection is still controversial [10, 12, 15]. Although Ulasoglu et al reported circulating ghrelin levels decreased in *H*. *pylori* eradicated subjects [16], a systematic review using 25 studies indicated that eradicating *H*. *pylori* did not influence circulating ghrelin levels [17]. In addition, there are few studies on the correlation between obestatin and *H*. *pylori* infection [10]. Other reports showed that plasma G/O ratio is related to inflammatory bowel diseases and obesity [12, 18, 19].

In this study, we aimed to investigate plasma ghrelin/obestatin levels in relation to the presence of *H*. *pylori* infection and subtypes of FD in elderly patients with FD. We also aimed to analyze the correlation between histological findings and plasma ghrelin/obestatin levels.

## Materials and methods

### Patients

Ninety-two patients aged more than 60 years diagnosed with FD between 2011 and 2012 were prospectively enrolled in this study. Both male and female subjects were included in the study. Clinical symptoms investigated include postprandial fullness, epigastric pain, epigastric burning, nausea, vomiting, and belching based on Rome III criteria [20]. All subjects underwent esophagogastroduodenoscopy after overnight fasting to rule out structural diseases such as erosive esophagitis, peptic ulcers, and gastric cancer. Patients aged below 60 years and those with structural diseases and history of gastric surgery and those using nonsteroidal anti-inflammatory drugs were excluded from the study. According to the Rome III criteria, patients diagnosed with FD were divided into two subtypes: epigastric pain syndrome (EPS) and PDS. We calculated the body mass index of all subjects during questionnaire completion. All endoscopies were performed by an experienced endoscopist. This study was approved by the institutional review board of Seoul Metropolitan Government Seoul National University Boramae Medical Center and was conducted according to the Declaration of Helsinki. Written informed consent was obtained from all patients who were enrolled in this study.

### *Helicobacter pylori* tests and histology

Mucosal samples obtained endoscopically from the antrum and corpus of the stomach were tested for presence of *H*. *pylori* infection based on histological demonstration of *H*. *pylori* (using modified Giemsa stain) or the Campylobacter-like organism (CLO) test (Institute of Immunology CO. Ltd., Tokyo, Japan). Positivity of one or both tests denotes infection with *H*. *pylori*.

The gastric mucosal biopsies were also examined under hematoxylin and eosin (H&E) staining for degree of inflammatory cell infiltration, gastric atrophy, and intestinal metaplasia by two experienced histopathologists. For each patient, tissue samples were taken from the antrum and corpus of the stomach. Biopsy tissues were fixed in 10% buffered formalin for 12 hours at room temperature, and dehydrated with a graded series of ethanol before being embedded in paraffin. Then, tissue sections were serially cut at 5 μm and 4 serial-cut sections were mounted on glass slides. After the deparaffinization and rehydration, H&E stain and modified Giemsa stain were performed. Twenty high power fields were observed (x400) on each section. The degree of inflammatory cell infiltration, gastric atrophy, intestinal metaplasia, and *H*. *pylori* density were evaluated using the updated Sydney system scores (0 = none, 1 = mild, 2 = moderate, and 3 = marked) [21]. Subjects were then divided into two groups according to the degree of gastric atrophy (atrophy vs. no atrophy), and variables were compared between the groups. Dichotomization was similar to that for intestinal metaplasia (intestinal metaplasia vs. no intestinal metaplasia).

### Measurement of plasma ghrelin and obestatin levels

After overnight fasting for 12 h, blood samples were drawn into chilled polypropylene tubes containing ethylene-diaminetetraacetic acid and aprotinin on the day of esophagogastroduodenoscopy and were kept immediately on ice. After immediate centrifugation at 1,600*g* for 15 minutes at 4°C, plasma samples were stored at -70°C until further use. Samples underwent acidification before peptides extraction according to the manufacturer’s instructions. Plasma total ghrelin levels were measured with commercially available ghrelin enzyme immunoassay (EIA) kits (Phoenix Pharmaceuticals, Brulingame, CA, USA). Plasma obestatin levels were measured using commercially available YK231 human obestatin EIA kits (Yanaihara Institute Inc., Shizuoka, Japan).

### Statistical methods

Descriptive statistics (means, standard deviations, and percentages) were calculated to characterize the patients. Comparisons between the groups were made using the Student’s *t*-test for continuous variables and the chi-square test for categorical variables. Histopathological scores were compared between the groups using the Mann-Whitney *U* test. A *P* value less than 0.05 was considered statistically significant. All statistical analyses were performed using SPSS version 20.0 (IBM Corp., Armonk, NY, USA).

## Results

A total of 92 patients who were diagnosed with FD and were older than 60 years (median age, 69.4; range 60–88 years) were included in this study (S1 File). Among the subjects, 43 were negative and 49 were positive for *H*. *pylori* infection. Body mass index, age, and the proportion of males did not vary according to *H*. *pylori* positivity. *H*. *pylori* activity and infiltration with neutrophils and mononuclear cells were significantly higher in the *H*. *pylori–*positive group. However, the presence of symptoms and the subtypes of FD between the groups were not significantly different (Table 1). Additionally, plasma total ghrelin and obestatin levels did not vary significantly between the *H*. *pylori*–positive and *H*. *pylori*–negative groups (Table 2). Since plasma total ghrelin and obestatin levels were not in normal distribution, logarithmic transformation was used. After logarithmic transformation, Pearson’s correlation analyses were performed between BMI, age and logarithmic transformed plasma ghrelin/obestatin levels, which showed no significant correlation (data not shown).

**Table 1:** Demographic, clinical, and pathological characteristics of the patients.

Variables	*H*.*pylori* negative	*H*.*pylori* positive	*P* value
	(N = 43)	(N = 49)	
Male, n (%)	17 (39.5)	21 (42.9)	0.747
Age (yr)	70.4 ± 7.2	68.6 ± 5.7	0.174
BMI (kg/m2)	22.8 ± 2.7	22.3 ± 2.3	0.374
**Bothersome symptoms (Number of subjects)**			
Epigastric pain	20 (46.5)	24 (49.0)	0.813
Epigastric burning	21 (48.8)	31 (63.3)	0.164
Postprandial fullness	34 (79.1)	43 (87.8)	0.261
Nausea	18 (41.9)	15 (30.6)	0.262
Vomiting	9 (20.9)	7 (14.3)	0.402
Belching	30 (69.8)	39 (79.6)	0.278
**Subtypes of FD**			
EPS	10 (23.3)	15 (30.6)	0.429
PDS	33 (76.7)	34 (69.4)	
**Antrum**			
*H*. *pylori* density	0	0.80 ± 0.84	<0.001
Neutrophil infiltration	0.16 ± 0.43	1.14 ± 0.96	<0.001
Mononuclear cell infiltration	1.26 ± 0.44	1.88 ± 0.48	<0.001
Intestinal metaplasia	0.50 ± 0.80	0.66 ± 0.92	0.384
Atrophy	0.52 ± 0.80	0.55 ± 0.77	0.884
**Body**			
*H*. *pylori* density	0	1.12 ± 0.88	<0.001
Neutrophil infiltration	0.21 ± 0.52	1.47 ± 0.98	<0.001
Mononuclear cell infiltration	1.14 **±** 0.35	1.84 ± 0.55	<0.001
Intestinal metaplasia	0.22 ± 0.65	0.17 ± 0.48	0.658
Atrophy	0.15 ± 0.50	0.16 ± 0.44	0.892

Values are presented as number (%) or mean±SD.

Multiple answers are allowed for bothersome symptoms

Pathological values correspond to Sydney scores and *P* values are calculated using linear by linear association test

EPS, epigastric pain syndrome; PDS, postprandial distress syndrome

**Table 2:** Comparison of plasma ghrelin and obestatin levels.

Variables	*H*.*pylori* negative	*H*.*pylori* positive	*P* value
	(N = 43)	(N = 49)	
Plasma ghrelin (pg/mL, log transformed)	6.75 ± 0.29	6.65 ± 0.36	0.130
Plasma obestatin (pg/mL, log transformed)	6.07 ± 0.20	6.07 ± 0.23	0.888
Plasma ghrelin/obestatin ratio	2.10 ± 0.71	1.93 ± 0.89	0.318

Values are presented as mean±SD.

Plasma ghrelin/obestatin ratio was calculated without logarithmic transformation.

Body mass index, age, and the proportion of males did not vary according to the subtypes of FD. Variables compared between the EPS and PDS groups did not demonstrate significant differences in the plasma ghrelin and obestatin levels. In addition, Histopathological findings did not differ between the groups (Table 3).

**Table 3:** Demographic, clinical and pathological characteristics of the patients according to subtypes of FD.

Variables	EPS	PDS	*P* value
	(N = 25)	(N = 67)	
Male, n (%)	12 (48.0)	26 (38.8)	0.426
Age (yr)	69.6 ± 8.1	69.4 ± 5.9	0.874
BMI (kg/m2)	22.1 ± 2.5	22.7 ± 2.5	0.366
Plasma ghrelin (pg/mL, log transformed)	6.76 ± 0.39	6.67 ± 0.30	0.238
Plasma obestatin (pg/mL, log transformed)	6.08 ± 0.15	6.07 ± 0.24	0.710
Plasma ghrelin/obestatin ratio	2.12 ± 1.00	1.97 ± 0.73	0.443
**Antrum**			
*H*. *pylori* density	0.49 ± 0.78	0.43 ± 0.74	0.608
Neutrophil infiltration	0.75 ± 0.91	0.70 ± 0.91	0.771
Mononuclear cell infiltration	1.67 ± 0.55	1.60 ± 0.56	0.777
Intestinal metaplasia	0.61 ± 0.87	0.60 ± 0.87	0.914
Atrophy	0.46 ± 0.70	0.56 ± 0.79	0.121
**Body**			
*H*. *pylori* density	0.75 ± 0.94	0.61 ± 0.87	0.058
Neutrophil infiltration	1.07 ± 1.06	0.91 ± 1.01	0.043
Mononuclear cell infiltration	1.54 ± 0.54	1.54 ± 0.59	0.662
Intestinal metaplasia	0.20 ± 0.56	0.18 ± 0.54	0.860
Atrophy	0.16 ± 0.49	0.13 ± 0.42	0.678

Values are presented as number (%) or mean±SD.

Plasma ghrelin/obestatin ratio was calculated without logarithmic transformation.

Pathological values correspond to Sydney scores and *P* values are calculated using linear by linear association test

EPS, epigastric pain syndrome; PDS, postprandial distress syndrome; FD, functional dyspepsia

Body mass index, age, and the proportion of males did not vary according to histopathological findings. A comparison between subjects with and without gastric atrophy did not show significant differences in plasma ghrelin and obestatin levels. In contrast, when subjects with and without intestinal metaplasia were compared, we found that patients with intestinal metaplasia had significantly lower plasma ghrelin levels and G/O ratio. To investigate gender effect, data were analyzed according to gender. Male subjects with intestinal metaplasia had significantly lower plasma ghrelin levels (Table 4). To assess gender effect or gender-by-subgroup interaction, we performed the analysis of covariance (ANCOVA). In the ANCOVA analyses, age and BMI were adjusted as confounding factors. Group effect was significant in males but not in females (Table 5).

**Table 4:** Clinical characteristics of the patients according to pathological findings.

Variables	Metaplasia	No metaplasia	*P* value	Atrophy	No atrophy	*P* value
	(N = 37)	(N = 55)		(N = 25)	(N = 57)	
Male, n (%)	19 (51.4)	19 (34.5)	0.108	13 (52.0)	22 (38.6)	0.259
Age (yr)	70.0 ± 6.1	69.1 ± 6.8	0.517	69.8 ± 6.9	69.3 ± 6.4	0.749
BMI (kg/m2)	22.7 ± 2.8	22.4 ± 2.3	0.623	22.6 ± 3.1	22.3 ± 2.2	0.567
Both gender						
Plasma ghrelin (pg/mL, log transformed)	6.59 ± 0.29	6.77 ± 0.34	0.010	6.60 ± 0.28	6.74 ± 0.35	0.081
Plasma obestatin (pg/mL, log transformed)	6.06 ± 0.12	6.08 ± 0.26	0.537	6.01 ± 0.07	6.09 ± 0.25	0.051
Plasma ghrelin/obestatin ratio	1.78 ± 0.54	2.17 ± 0.92	0.012	1.87 ± 0.53	2.10 ± 0.91	0.150
Male						
Plasma ghrelin (pg/mL, log transformed)	6.54 ± 0.30	6.84 ± 0.37	0.008	6.58 ± 0.32	6.80 ± 0.37	0.083
Plasma obestatin (pg/mL, log transformed)	6.00 ± 0.08	6.08 ± 0.31	0.320	5.99 ± 0.06	6.07 ± 0.29	0.241
Plasma ghrelin/obestatin ratio	1.81 ± 0.61	2.41 ± 1.16	0.051	1.90 ± 0.66	2.32 ± 1.12	0.233
Female						
Plasma ghrelin (pg/mL, log transformed)	6.64 ± 0.27	6.73 ± 0.32	0.325	6.63 ± 0.23	6.70 ± 0.33	0.463
Plasma obestatin (pg/mL, log transformed)	6.11 ± 0.14	6.08 ± 0.23	0.635	6.04 ± 0.08	6.10 ± 0.23	0.201
Plasma ghrelin/obestatin ratio	1.75 ± 0.48	2.04 ± 0.75	0.086	1.83 ± 0.37	1.97 ± 0.74	0.412

Plasma ghrelin/obestatin ratio was calculated without logarithmic transformation.

**Table 5:** Analysis of covariance according to gender.

Response variable		Variable	*F* statistic	*P* value		Variable	*F* statistic	*P* value
Plasma ghrelin (pg/mL, log transformed)	All	Sex	1.711	0.194	All	Sex	0.105	0.747
		Metaplasia	9.710	0.003		Atrophy	3.131	0.080
		Sex, Metaplasia	2.767	0.100		Sex, Atrophy	0.380	0.539
	Male	Metaplasia	7.898	0.008	Male	Atrophy	2.544	0.120
	Female	Metaplasia	1.276	0.264	Female	Atrophy	1.472	0.231
Plasma obestatin (pg/mL, log transformed)	All	Sex	0.057	0.811	All	Sex	0.388	0.535
		Metaplasia	1.386	0.243		Atrophy	0.856	0.357
		Sex, Metaplasia	1.560	0.215		Sex, Atrophy	0.024	0.877
	Male	Metaplasia	0.975	0.331	Male	Atrophy	0.957	0.335
	Female	Metaplasia	0.367	0.548	Female	Atrophy	1.903	0.174
Plasma ghrelin/obestatin ratio	All	Sex	2.166	0.145	All	Sex	0.956	0.331
		Metaplasia	5.745	0.019		Atrophy	1.672	0.200
		Sex, Metaplasia	0.853	0.358		Sex, Atrophy	0.349	0.556
	Male	Metaplasia	3.817	0.059	Male	Atrophy	1.082	0.306
	Female	Metaplasia	2.826	0.099	Female	Atrophy	0.568	0.455

Plasma ghrelin/obestatin ratio was calculated without logarithmic transformation.

## Discussion

In this study, we investigated whether plasma total ghrelin and obestatin levels are related to histopathological findings, *H*. *pylori* infection, and/or FD subtypes in elderly patients with FD. While our results showed that plasma total ghrelin levels and G/O ratio did not differ between subjects with and without gastric atrophy, we found that these were significantly related to intestinal metaplasia, a known precursor of gastric cancer. This study is the first to demonstrate such an association.

A previous study showed that ghrelin correlated with the topographic distribution of gastric atrophy, and histological glandular atrophy scores according to updated Sydney system were related to plasma ghrelin levels [22]. This study by Ikeda et al suggested lower ghrelin levels are related to more severe gastric atrophy. However, the study population was not limited to the older age group. Reports have also shown that circulating ghrelin levels decrease with age; however, the causes of this tendency are not clear [23]. Another study including elderly patients showed an association between gastric atrophy and ghrelin expression, although intestinal metaplasia was not evaluated in this study [24]. In our study with elderly patients, intestinal metaplasia was the only variable significantly related to the decrease in plasma ghrelin levels. Plasma G/O ratio also showed a correlation with intestinal metaplasia.

Ulasoglu et al. reported that obestatin levels are increased in the *H*. *pylori*–eradicated group [16]. Gao et al’s study showed that ghrelin levels and G/O ratio were lower in the group with *H*. *pylori* infection [10]. However, while these two studies addressed obestatin levels, they did not include histological findings, and their study population was not restricted to those with advanced age. In contrast to our study, Gao et al’s study showed circulating ghrelin and G/O ratios were lower in *H*. *pylori*-positive group than *H*. *pylori*-negative group. Although total ghrelin levels were assessed using the same RIA kit (Phoenix Pharmaceuticals) as in our study, Gao et al’s study did not assess histological atrophic scores. In our study which analyzed histological findings, *H*. *pylori* infection did not affect plasma ghrelin levels, but intestinal metaplasia influenced plasma ghrelin levels and G/O ratios. Previous reports showed that inflammatory bowel diseases and obesity were related to the circulating G/O ratio [12, 18, 19]. Our study did not reveal a significant correlation between obestatin levels and histological findings, but the G/O ratio was found to be related to intestinal metaplasia.

According to Osawa and Isomoto, patients with gastric atrophy due to *H*. *pylori* infection have reduced total plasma ghrelin [25, 26]. However, in those without gastric atrophy, *H*. *pylori* did not affect the ghrelin levels [12]. From these results, it can be presumed that the inflammation or atrophy resulting from *H*. *pylori* infection induces the loss of ghrelin-producing cells, which in turn results in the decrease in plasma ghrelin levels. As atrophy progresses to intestinal metaplasia with age, elderly subjects have a higher risk of intestinal metaplasia, a well-known precursor of gastric cancer [14, 27]. In Gao et al’s study, total ghrelin levels were estimated, and plasma ghrelin levels, obestatin levels, and G/O ratio were all lower in patients with gastric atrophy [11]. Of interest, our study showed significant association between ghrelin levels, G/O ratio, and intestinal metaplasia, but not gastric atrophy. This can be attributed to the small sample size, because tendency towards lower plasma ghrelin levels and G/O ratios were observed in subjects with gastric atrophy than those without gastric atrophy. Additionally, our study showed that only plasma ghrelin levels and G/O ratio were lower in the group with intestinal metaplasia (plasma obestatin levels did not differ significantly). This could be partially explained by the fact that obestatin is produced in multiple organs [28–30]. Studies have reported that while plasma ghrelin levels decreased by 65% after gastrectomy [31], plasma obestatin levels did not [32]. These findings suggest that the main source of obestatin may be different from that of ghrelin, although both are produced in neuroendocrine cells of oxyntic gland and are derived from the same prohormone [33]. Ghrelin gene-derived mRNA transcripts that do not code for ghrelin, but encode only obestatin have been described [34]. As plasma ghrelin levels are sexually dimorphic, analyses were performed to investigate gender effect (Tables 4 and 5). Males with intestinal metaplasia had lower plasma ghrelin levels. This may be explained by the fact that fluctuating levels of estrogen affect plasma ghrelin levels [35].

There have been reports that described the association between *H*. *pylori* and ghrelin levels [15, 17]. However, other studies reported that it was not *H*. *pylori*, but histological atrophy scores or the extent of gastric mucosal atrophy, which was related to plasma ghrelin levels [22, 36]. Histological changes such as intestinal metaplasia are presumed to be related to the loss of ghrelin-producing cells, which in turn affects the plasma ghrelin levels. Notwithstanding the fact that ghrelin levels were measured in the circulation and not directly in the stomach, since most portion of ghrelin is produced in gastric cells [31], the results of our study seem to support these findings.

Ghrelin is known to be related to gastric motility, which is the pathophysiology of PDS rather than EPS [37]. Accordingly, in studies analyzing the correlation between FD and ghrelin, the FD patients studied were mostly those with PDS or dysmotility-like FD [7–9]. We compared ghrelin and obestatin levels between the PDS and EPS groups but could not detect significant differences. This applied to the histological findings as well.

Our study has some limitations. First, as we did not include a healthy control group, comparison between FD patients and a healthy population was not possible. Second, the correlation found between plasma ghrelin, G/O ratio, and atrophic gastritis in this study may be due to the small sample size. Third, ghrelin levels were measured in the circulation, and immunohistochemistry using gastric mucosal tissue was not performed in our study. Finally, gastric mucosal biopsies were sampled from only the antrum and corpus of the stomach. Therefore, the histopathological examination of these tissues may not accurately represent the degree of atrophy or intestinal metaplasia of the entire gastric mucosa since gastric atrophic changes generally progress from the antrum to the lesser curvature of the gastric body, and finally throughout the entire stomach [38]. In contrast to the location of gastric atrophy, gastric ghrelin is produced in the chromogranin A-immunoreactive X/A-like endocrine cells located in the mucosal layer of the fundus [39].

In conclusion, plasma ghrelin levels and plasma G/O ratio in elderly patients with FD were found to be significantly reduced in patients with intestinal metaplasia. The results of this study imply that plasma ghrelin levels and G/O ratio are related to intestinal metaplasia. As intestinal metaplasia is a known precursor of gastric cancer, measurement of these parameters may be helpful in predicting the development of gastric cancer.

## Supporting information

S1 File Ghrelin obestatin data file. (XLSX)
